# Supplementary material for: Detectability of Iodine in Mediastinal Lesions on Photon Counting CT: A Phantom Study
Source: Diagnostics (Basel). 2025 Mar 11;15(6):696. doi: 10.3390/diagnostics15060696 (PMC11941654; doi:10.3390/diagnostics15060696)
Supplement: Supplementary file 1 [file diagnostics-15-00696-s001.zip › diagnostics-3428539-supplementary.pdf]

**Table S1.** Mean CT-values of the just detectable insert diameters with iodine concentration on conventional computed tomography (CCT) and photon-counting CT (PCCT) as a function of virtual monochromatic image (VMI) reconstruction, dose level, slice thickness, and matrix size.

| | CT system | VMI (keV) | Dose % | slice thick-ness (mm) | Matrix size | Diameter (mm) | Concen-tration (mg/ml) | Mean CT-value (HU) |
|---|---|---|---|---|---|---|---|---|
| **Reference CCT** | CCT | | 100 | 1 | 512 | 1 | 27.5 | 302 |
| | CCT | | 100 | 1 | 512 | 3 | 11.2 | 105 |
| | CCT | | 100 | 1 | 512 | 5 | 4.54 | 75 |
| | CCT | | 100 | 1 | 512 | 8 | 1.43 | 34 |
| | CCT | | 100 | 1 | 512 | 12 | 1.43 | 37 |
| **Variation of VMI** | PCCT | 40 | 100 | 1 | 512 | 1 | 27.5 | 980 |
| | PCCT | 40 | 100 | 1 | 512 | 3 | 1.43 | 119 |
| | PCCT | 40 | 100 | 1 | 512 | 5 | 0.238 | 81 |
| | PCCT | 40 | 100 | 1 | 512 | 8 | 0.238 | 79 |
| | PCCT | 40 | 100 | 1 | 512 | 12 | 0.238 | 80 |
| | PCCT | 50 | 100 | 1 | 512 | 1 | 27.5 | 642 |
| | PCCT | 50 | 100 | 1 | 512 | 3 | 1.43 | 115 |
| | PCCT | 50 | 100 | 1 | 512 | 5 | 0.238 | 67 |
| | PCCT | 50 | 100 | 1 | 512 | 8 | 0.238 | 75 |
| | PCCT | 50 | 100 | 1 | 512 | 12 | 0.238 | 70 |
| | PCCT | 60 | 100 | 1 | 512 | 1 | 27.5 | 437 |
| | PCCT | 60 | 100 | 1 | 512 | 3 | 4.54 | 96 |
| | PCCT | 60 | 100 | 1 | 512 | 5 | 1.43 | 59 |
| | PCCT | 60 | 100 | 1 | 512 | 8 | 1.43 | 60 |
| | PCCT | 60 | 100 | 1 | 512 | 12 | 1.43 | 58 |
| | PCCT | 70 | 100 | 1 | 512 | 1 | 27.5 | 367 |
| | PCCT | 70 | 100 | 1 | 512 | 3 | 4.54 | 81 |
| | PCCT | 70 | 100 | 1 | 512 | 5 | 1.43 | 40 |
| | PCCT | 70 | 100 | 1 | 512 | 8 | 1.43 | 35 |
| | PCCT | 70 | 100 | 1 | 512 | 12 | 1.43 | 36 |
| **Variation of dose** | PCCT | 50 | 34 | 1 | 512 | 1 | 27.5 | 642 |
| | PCCT | 50 | 34 | 1 | 512 | 3 | 4.54 | 177 |
| | PCCT | 50 | 34 | 1 | 512 | 5 | 4.54 | 180 |
| | PCCT | 50 | 34 | 1 | 512 | 8 | 1.43 | 108 |
| | PCCT | 50 | 34 | 1 | 512 | 12 | 1.43 | 92 |
| | PCCT | 50 | 42 | 1 | 512 | 1 | 27.5 | 692 |
| | PCCT | 50 | 42 | 1 | 512 | 3 | 4.54 | 122 |
| | PCCT | 50 | 42 | 1 | 512 | 5 | 1.43 | 64 |
| | PCCT | 50 | 42 | 1 | 512 | 8 | 1.43 | 74 |

| | | | | | | | | |
|---|---|---|---|---|---|---|---|---|
| | PCCT | 50 | 42 | 1 | 512 | 12 | 1.43 | 65 |
| | PCCT | 50 | 52 | 1 | 512 | 1 | 27.5 | 586 |
| | PCCT | 50 | 52 | 1 | 512 | 3 | 4.54 | 145 |
| | PCCT | 50 | 52 | 1 | 512 | 5 | 1.43 | 72 |
| | PCCT | 50 | 52 | 1 | 512 | 8 | 1.43 | 63 |
| | PCCT | 50 | 52 | 1 | 512 | 12 | 1.43 | 67 |
| **Variation of slice thickness** | PCCT | 50 | 100 | 0.2 | 512 | 1 | 27.5 | 650 |
| | PCCT | 50 | 100 | 0.2 | 512 | 3 | 11.2 | 269 |
| | PCCT | 50 | 100 | 0.2 | 512 | 5 | 4.54 | 115 |
| | PCCT | 50 | 100 | 0.2 | 512 | 8 | 1.43 | 46 |
| | PCCT | 50 | 100 | 0.2 | 512 | 12 | 1.43 | 43 |
| | PCCT | 50 | 100 | 0.4 | 512 | 1 | 27.5 | 647 |
| | PCCT | 50 | 100 | 0.4 | 512 | 3 | 4.54 | 81 |
| | PCCT | 50 | 100 | 0.4 | 512 | 5 | 1.43 | 56 |
| | PCCT | 50 | 100 | 0.4 | 512 | 8 | 1.43 | 58 |
| | PCCT | 50 | 100 | 0.4 | 512 | 12 | 0.238 | 49 |
| **Variation of matrix size** | PCCT | 50 | 100 | 1 | 768 | 1 | 27.5 | 562 |
| | PCCT | 50 | 100 | 1 | 768 | 3 | 1.43 | 105 |
| | PCCT | 50 | 100 | 1 | 768 | 5 | 0.238 | 64 |
| | PCCT | 50 | 100 | 1 | 768 | 8 | 0.238 | 59 |
| | PCCT | 50 | 100 | 1 | 768 | 12 | 0.238 | 64 |
| | PCCT | 50 | 100 | 1 | 1024 | 1 | 11.2 | 297 |
| | PCCT | 50 | 100 | 1 | 1024 | 3 | 0.238 | 57 |
| | PCCT | 50 | 100 | 1 | 1024 | 5 | 0.238 | 57 |
| | PCCT | 50 | 100 | 1 | 1024 | 8 | 0.238 | 53 |
| | PCCT | 50 | 100 | 1 | 1024 | 12 | 0.238 | 51 |
